# Impetiginous Cutaneous Leishmaniasis after COVID-19 Infection in a Patient with Poor Cardiac Profile: A Case Report and Literature Review

**DOI:** 10.3390/tropicalmed8090443

**Published:** 2023-09-10

**Authors:** Hend Alotaibi, Abdulelah Aldossari, Sultan Alnasser

**Affiliations:** 1 Department of Dermatology, College of Medicine, King Saud University, Riyadh 11472, Saudi Arabia; dr.alotaibihm@gmail.com; 2 College of Medicine, King Saud University, Riyadh 11472, Saudi Arabia; sultanalnasser70@gmail.com

**Keywords:** cutaneous leishmaniasis, COVID-19, presentation, association, treatment, inactive lesions

## Abstract

Cutaneous leishmaniasis incidence has been rising in the past couple of decades. Standard therapy often includes antileishmanial drugs; however, due to their low safety and toxicity threshold, alternative treatments are being investigated. The association between COVID-19 and cutaneous leishmaniasis remains unclear and exploring this connection may offer crucial insights into the pathophysiology of and treatment strategies for infected patients. In this article, we describe a case of a male patient with a history of cardiac and other comorbidities who presented with cutaneous leishmaniasis in the form of impetigo-like skin lesions after being infected with COVID-19. Due to the patient’s poor cardiac profile, sodium stibogluconate was not used and an alternative therapeutic approach was employed. The patient was treated with oral terbinafine, cryotherapy on specific lesions, and a course of cephalexin. Following the course of treatment and subsequent follow-up, the patient exhibited complete resolution and healing of the lesions with scarring, and no active lesions or recurrence were observed. This case highlights the potential for alternative treatment strategies for cutaneous leishmaniasis in patients with comorbidities and emphasizes the importance of further research to better understand the link between COVID-19 and cutaneous leishmaniasis.

## 1. Introduction

Leishmaniasis is caused by Leishmania protozoa, a spectrum of protozoan infections spread by phlebotomine sandflies to mammals, including humans. The burden of leishmaniasis is significant, with the Global Burden of Disease (GBD) study of 2019 estimating an annual occurrence of approximately 498,000 to 862,000 new cases across all forms of the disease. This results in a considerable number of fatalities, with up to 18,700 reported every year, and a loss of approximately 1.6 million disability-adjusted life years (DALYs) [1]. It is worth noting that leishmaniasis was previously considered one of the most neglected tropical diseases (NTDs) in terms of resources allocated to its diagnosis, treatment, and control. It contributes to 4% of the global NTD DALY burden and accounts for 5.5% of NTD-associated mortalities worldwide [2]. However, these figures may underestimate the true impact of leishmaniasis due to under-reporting. Several studies suggest that the actual number of DALYs lost could be up to ten times higher than the current estimates [3,4,5].

Depending on the species that caused the infection, a Leishmania parasite infection might have one of three clinical presentations. Localized cutaneous leishmaniasis (CL) is the first type and it can result in a single or multiple skin ulcers, satellite lesions, or nodular lymphangitis. The second form of leishmaniasis is CL with mucosal involvement (MCL). Systemic visceral leishmaniasis (VL), the third type, can have catastrophic consequences if left untreated as it affects internal organs such as the bone marrow, liver, and spleen [6].

CL is characterized by skin lesions that can present in various forms, including papules, nodules, ulcers, or plaques, depending on the causative species and host immune response [7]. Even though CL is minor and not life-threatening condition, its disfiguring lesions and scars can have a substantial detrimental influence on a person’s social and psychological aspects, causing anxiety, depression, a decline in body satisfaction, and a poor quality of life [8,9,10].

CL generally has a self-limiting course. Specific antileishmanial therapy may hasten remission, shorten the infectivity duration, minimize the chance of recurrence (particularly in the case of subsequent immunological impairment), and lower the risk for metastatic dissemination [11].

Therapeutic interventions in CL are based on the disease severity, which is determined by the number and size of the lesions, presence of mucosal involvement, host immunological status, geographical location, and the causing species [12,13].

The recent health crisis caused by the rapid and global spread of SARS-CoV-2 leading to COVID-19 disease is an ongoing pandemic with severe consequences that continue to impact many countries worldwide. In addition to the significant mortality and morbidity burden brought on by COVID-19, the pandemic has had several direct and indirect effects on the incidence rates of many communicable and noncommunicable diseases; one of which is leishmaniasis [14].

In this article, we present a case of a patient from the Middle East who developed impetiginous cutaneous leishmaniasis after recovering from COVID-19. The patient has a history of deteriorating cardiac profile, preventing him from receiving the classical antileishmanial regimens.

## 2. Case Presentation

A 76-year-old Syrian male, known to have ischemic cardiomyopathy with an ejection fraction of 25–30% requiring an implantable cardioverter defibrillator, treated bladder cancer, hypothyroidism, and interstitial lung disease, presented to the emergency department on 20 January 2022 with a 4-month history of multiple painful erythematous skin lesions that developed while he was in Syria which then progressed over time. Before developing the skin lesions, the patient reported having experienced a COVID-19 infection and required admission and hospitalization for two weeks. The patient noticed formation of skin lesions two weeks after that.

According to the patient, the lesions first started 4 months ago as small erythematous papules over the upper extremities that progressed slowly in size, with significant worsening over the past 15 days associated with tenderness. Lesions were not associated with any discharges or bleeding. The patient was afebrile and denied any history of trauma, insect bites, or similar presentations in the past.

**Figure 1:**
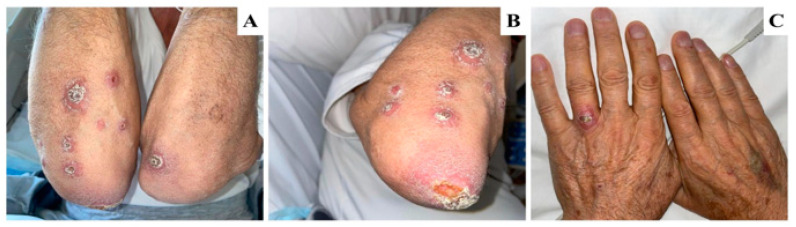
(**A**–**C**). Initial presentation of cutaneous lesions. (**A**) Multiple erythematous plaques over the extensor surface of forearms. (**B**) Erythematous plaques with central hemorrhagic crust over the right elbow. (**C**) Erythematous plaques on left hand.

Skin examination showed multiple erythematous plaques with central hemorrhagic crustations over bilateral upper extremities, mainly involving the extensor aspects and the elbow area. No other areas were involved (Figure 1). The first impression was infectious processes including ecthyma, which is a deep type of impetigo or a neoplastic etiology of cutaneous metastasis. A swab was obtained from the lesion over the right elbow and sent for bacterial culture and sensitivity, clinical samples for smears were collected, and a skin biopsy was taken from the lesions over the right arm and sent for H/E staining to rule out cutaneous metastasis. The patient was instructed to apply topical ketoconazole and fusidic acid ointment over the lesions and clean the lesions with normal saline and gauze twice daily for one week until the results of the skin biopsy were ready.

**Figure 2:**
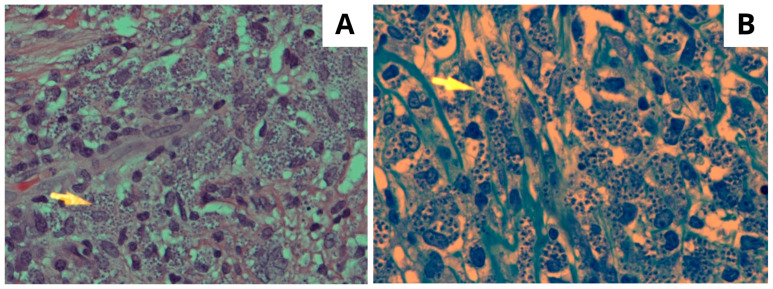
(**A**) High power microscopic view of the affected dermis showing numerous foamy and vacuolated macrophages containing Leishman–Donovan bodies (arrowhead) H/E stain ×400. (**B**) The Giemsa-stained section highlighting numerous classic Leishman–Donovan bodies within histiocytic cells. Giemsa special stain ×600.

Histopathological examination (Figure 2) showed epidermal ulceration with a crust formation. In the dermis, there was necrotizing ill-defined granuloma with heavy inflammatory infiltrate including lymphocytes, plasma cells, neutrophils, and histiocytes. The histiocytes contained small round to oval organisms with bar-shaped paranuclear kinetoplasts. The Giemsa stain was positive. The GMS and PAS stains for fungal organisms were negative. PCR was not performed due to unavailability. These features were consistent with CL, and the diagnosis was established.

**Figure 3:**
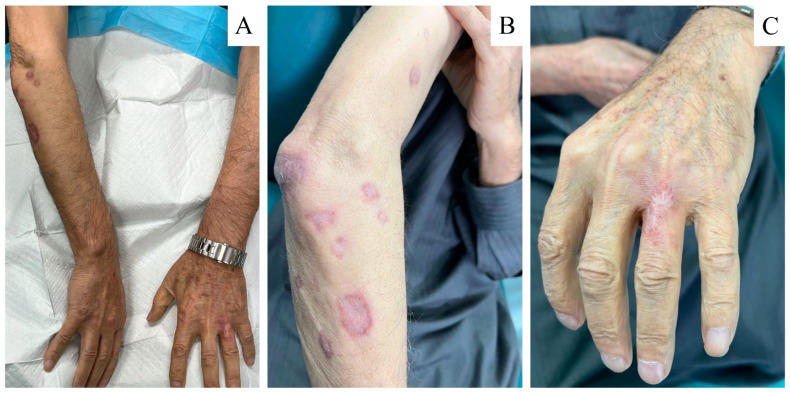
(**A**–**C**) Healed cut lesions after treatment with oral terbinafine and cryotherapy. (**A**) complete resolution of the lesions bilaterally. (**B**) Inactive lesions with scarring over the right forearm. (**C**) Healed lesion over the left hand.

Taking into consideration the poor cardiac profile of the patient, sodium stibogluconate was not an option due to its risk of cardiotoxicity and arrhythmia. The patient was given oral terbinafine 250 mg twice daily for 6 weeks and was to be followed up in 6 weeks. After 6 weeks, the patient reported no improvement. The patient was given another trial of oral terbinafine for 2 months. After 2 months, in the next visit, the patient reported moderate improvement and was instructed to continue on terbinafine for another 3 months. Cryotherapy was performed over seven non-crusted lesions over the right arm and one lesion over the left arm, and cephalexin 250 mg BID was prescribed for 7 days. Eight months after the initial treatment, the lesions showed complete resolution and healing with scarring of the affected area, and no active lesions or recurrence were observed (Figure 3).

## 3. A Brief Literature Review and Comments

Data from surveillance programs show that during the past 20 years, there has been an increase in the number of incidents of leishmaniasis reported worldwide [15]. Such increased incidences can be explained by a number of factors, including increased detection of CL linked to opportunistic illnesses (such as HIV/AIDS), poor vector or host control, the rise in antileishmanial drug resistance, and the implementation of better diagnosis and case notification systems [16,17,18].

CL is the prevailing form of leishmaniasis worldwide, and a mere ten countries are responsible for 75% of all cases. These nations include Algeria, Afghanistan, Brazil, Colombia, Costa Rica, Ethiopia, Iran, North Sudan, Peru, and Syria [19].

Though CL is not a fatal disease, it impacts the quality of life. As the World Health Organization defines health as consisting of physical, mental, and social well-being, it is clear that CL affects all three dimensions. CL has a significant effect on the quality of life and psychological impact both on patients with active disease and on patients with residual scars [8].

Immunologically, CL is marked by a robust type 1 T cell reaction to Leishmania antigens, resulting in the release of significant amounts of interferon-gamma (IFN-γ) and tumor necrosis factor (TNF) [20]. While an overabundance of IFN-γ and cytotoxic CD8+ T cells may contribute to the inflammation that leads to ulcer formation [21,22,23], the protective role of IFN-γ in CL is well recognized. Low IFN-γ levels are found in diffuse cutaneous leishmaniasis (DCL) patients infected with L. amazonensis [24]. In various forms of the disease, the microbicidal effects of IFN-γ are counteracted by interleukin-10 (IL-10) in vivo [20] or within macrophages in vitro [25,26].

Few studies have investigated the prophylactic effect of CL against COVID-19 and its protective mechanism [27]. Similar to the protective Th1 immune response against Leishmania, the protective antiviral immune response against SARS-CoV2 is dependent on the production of IFN-γ and the subsequent activation of NK and CD8+ T cells. Although it makes sense that immune activity against one of these infections would also provide protection from the other, the interaction of immune responses is far more complicated and the outcome is still undetermined.

In our case, it is conceivable that COVID-19 might have led to leishmaniasis that had been asymptomatic to reactivate. Repolarizing the Th1 immune response to overcome the virus may have caused the parasite to escape immune surveillance, resulting in symptomatic CL. As seen by numerous reports, COVID-19 has caused the reactivation of a number of chronic, asymptomatic infections brought on by viruses (such as VZV, EBV, CMV, HSV, HHV6, HBV) [28,29,30], protozoa [31,32], and fungi [33].

Two additional explanations might be considered. The treatment for COVID-19 in Syria could have influenced the emergence of CL lesions, possibly due to the use of systemic steroids or other immunosuppressive substances. The patient could have been infected with Leishmania parasites shortly before or after the hospitalization period, even though they did not recall any insect bites.

Morphologically, patients with localized CL can exhibit a variety of lesion forms; ulcerative lesions, which account for 90% of all cases, are the most common, followed by nodular and nodulo-ulcerative lesions [34]. Furthermore, the size of the lesions varies; smaller lesions (1–2 mm) are more common [35]. In some cases, disseminated lesions have been described as subcutaneous nodules, satellite papules, and subcutaneous indurations. Non-Saudis were more likely to develop such lesions [36]. This could be attributed to herd immunity that has grown as a result of repeated exposure to sand fly bites in disease-endemic areas. In 10% of cases, it has also been recorded that regional lymph nodes enlarge, and dissemination is achieved through lymphatics pathways in subcutaneous nodules associated with palpable enlarged lymphatics; this condition is known as sporotrichoid CL and has also been documented among Saudi patients [37,38]. Other uncommon clinical manifestations have also been described in the region, such as mycetoma-like lesions and chiclero ulcers, where the lesion involves the rim of the pinna [39].

Treatment challenges in CL arise from numerous factors affecting drug efficacy, such as lesion size, number, and appearance; disease duration before treatment; self-healing frequency and time; relapse and remission rates; frequency and severity of mucosal or diffuse involvement; immunosuppression; co-infections; prior anti-Leishmania treatment; and resistance to anti-Leishmania drugs [40]. Laboratory studies have reported acquired resistance to anti-Leishmania drugs for years, but clinical resistance has only recently been described. Monitoring resistance is currently controversial due to insufficient correlation between clinical and in vitro resistance and the need for understanding the resistance’s biochemical and molecular mechanisms [18].

The therapeutic approach is often determined by factors such as lesion location (e.g., face or joints) and the patient’s sex and age [41]. Other factors are intrinsic to the different *Leishmania* species [42]. An effective treatment in one geographical area for a specific organism may not work in another area or for a different organism in the same location. In these situations, efficacy depends not only on the *Leishmania* species but also on the individual’s response to the parasite and factors like immunity, variable clinical response to treatments, drug toxicity, drug resistance, HIV co-infection, and adherence [43]. Various authors have described numerous treatments for Old World cutaneous leishmaniasis (OWCL) [44,45,46]. However, several authors have noted the lack of well-controlled clinical trials [47,48,49,50]. Another issue is the limited availability of most of these drugs in rural and poorer areas where leishmaniasis is more prevalent [40].

Systemic treatments are typically administered to CL patients with large (≥5 cm), multiple (>5), or disseminated lesions; those with simple lesions involving cosmetically sensitive areas or joints; those with mucosal reactions, nodular lymphangitis, or lymphadenopathies; or those for whom local therapy has failed [43]. For immunosuppressed individuals, there is controversy. While some experts believe acquired or induced immunosuppression is a risk factor for developing mucosal leishmaniasis and recommend systemic treatment, others consider different treatments for the same population [51].

CL has few treatments available. In most regions, pentavalent antimonials (e.g., sodium stibogluconate, Pentostam, or meglumine antimoniate) continue to be the first line of treatment for CL. Miltefosine, pentamidine isethionate, amphotericin B, paromomycin, heat therapy, and cryotherapy are a few alternative treatment regimens [12,13].

Although sodium stibogluconate and meglumine antimoniate are being used extensively, there are concerns over their cost, their toxicity, and the emergence of drug resistance. Parenteral antimonial medications can have serious adverse effects that are typically dosage dependent, such as nausea, vomiting, diarrhea, skin rashes, dizziness, cardiac arrhythmia, hypotension, increased level of hepatic enzymes, and pain at site of injections [52].

Terbinafine, an antifungal from the allylamine group, inhibits ergosterol synthesis through the suppression of squalene epoxidase [53]. The primary strength of terbinafine can be attributed to the fact that the drug generally exhibits minimal side effects, with occasional reports of taste loss, hepatitis, erythema multiforme, Stevens–Johnson syndrome, toxic epidermal necrolysis, neutropenia, and pancytopenia [54,55,56,57,58]. In an experimental study conducted by Bahashwan et al. (2011) in Saudi Arabia, the efficacy of terbinafine on the Leishmania parasite was investigated. By comparing the effects of terbinafine on *L. major* between case and control groups, it was observed that the case group, where BALB/c mice were administered terbinafine, demonstrated a significant reduction in the size of the leishmanial lesions [59].

Farajzadeh et al. (2015) investigated the role of oral terbinafine on humans as an alternative option for the treatment of CL, and they showed good results compared to the standard therapy [60].

In contrast, Bezemer et al. (2021) conducted a comprehensive systematic review of 22 articles investigating the safety and efficacy of terbinafine in the treatment of CL. Notably, their findings revealed a presence of bias and inaccuracies attributed to inadequate study designs and a failure to attain the intended primary outcomes. This is especially noteworthy considering the positive results observed in in vitro studies [61].

Cryotherapy using liquid nitrogen has been employed to treat individual lesions by destroying infected tissue. As an effective, painless, low-cost method with minimal side effects, it is particularly suitable for children. Nonetheless, it is not appropriate for multiple or complex lesions [12].

Numerous studies highlight that combination therapy not only improves efficacy in treating CL but also decreases the dosage of each drug, reduces their adverse effects, shortens treatment duration, and lowers the risk of developing drug resistance [62,63,64,65]. Similarly, a significant rise in the cure rate (CR) of CL patients was observed when treated with a combination of meglumine antimoniate (MA) and cryotherapy in comparison to patients treated with either meglumine or cryotherapy alone [62,66].

It is also crucial to consider the potential for the spontaneous resolution of CL lesions over time. Some studies have shown that certain forms of CL can self-resolve without any treatment, although this usually occurs over a prolonged period and is not guaranteed for all cases [15,67]. The natural history of CL suggests that the immune system can eventually control and clear the infection, leading to the healing of lesions [68]. Therefore, when assessing the efficacy of any treatment modality like terbinafine, meglumine antimoniate, or combination therapies, it is imperative to differentiate the treatment-induced healing from the natural, self-limiting course of the disease. The prolonged follow-up in some studies could potentially skew data in favor of treatment efficacy when, in fact, spontaneous resolution could be a contributing factor. This aspect is particularly important in the design and interpretation of controlled trials aiming to evaluate the efficacy of new or existing treatments for CL [15].

## 4. Conclusions

Knowing that CL is a great imitator, especially in regions where it has been endemic, diagnosis might be challenging especially when the lesions present with various other differentials, including inflammatory, granulomatous, or neoplastic lesions; careful clinical examination and diagnostic approaches should be followed.

The majority of CL treatment trials have been poorly designed and reported, leaving little solid proof of potentially helpful therapies. Large, well-designed studies are required to assess the long-term effects of existing treatments, the recurrence rates of CL, and the risk of progression to mucosal disease.

Finally, with the rising COVID-19 pandemic, the immunological pathways of SARS-CoV-2 and their effects on other immune responses should be investigated.

## Data Availability

Data are reported in the current study and are available on request from the corresponding author. Data sharing is not applicable to this article as no datasets were generated or analyzed during the current study.
